# Supplementary material for: Construction of a Novel 3D Urinary Bladder Mucosa Model and Its Application in Toxicity Assessment of Arsenicals
Source: Toxics. 2025 Sep 29;13(10):828. doi: 10.3390/toxics13100828 (PMC12568184; doi:10.3390/toxics13100828)
Supplement: Supplementary file 1 [file toxics-13-00828-s001.zip › toxics-3859493-supplementary.pdf]

Construction of a novel 3D urinary bladder mucosa model and its application in toxicity assessment of arsenicals

Runjie Guo [1], Min Gi [1,*], Tohru Kiyono [2], Arpamas Vachiraarunwong [1], Shugo Suzuki [3], Masaki Fujioka [3], Guiyu Qiu [1], Kwanchanok Praseatsook [1], Yurina Kawamura [1], Anna Kakehashi [3], Ikue Noura [3], Xiaoli Xie [4] and Hideki Wanibuchi [1,3]

[1] Department of Environmental Risk Assessment, Osaka Metropolitan University Graduate School of Medicine, Osaka,554-8585, Japan; sy23105k@st.omu.ac.jp (R.G.); m22438g@omu.ac.jp (A.V.); si22394d@st.omu.ac.jp (G.Q.); kwanchanok_pa@cmu.ac.th (K.P.); st25261t@st.omu.ac.jp (Y.K.); z21515o@omu.ac.jp (H.W.)

[2] Project for Prevention of HPV-Related Cancer, Division of Collaborative Research and Development, Exploratory Oncology Research and Clinical Trial Center, National Cancer Center, Kashiwa, 277-8577, Japan; tkiyono@east.ncc.go.jp

[3] Department of Molecular Pathology, Osaka Metropolitan University Graduate School of Medicine, Osaka,554-8585, Japan; f21049w@omu.ac.jp (S.S.); b21405o@omu.ac.jp (M.F.); anna-k@omu.ac.jp (A.K.); sx23713o@st.omu.ac.jp (I.N.)

[4] Department of Toxicology, School of Public Health, Southern Medical University (Guangdong Provincial Key Laboratory of Tropical Disease Research), Guangzhou,510515, China; xiexiaoli1999@126.com

[*] Correspondence: o21773j@omu.ac.jp

Supplementary material:

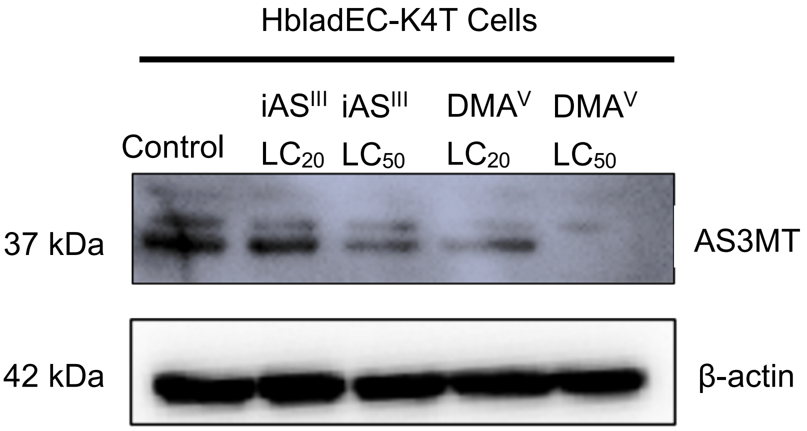

Supplementary Figure S1. Western blot analysis of AS3MT expression in HBladEC-K4T cells exposed to iAs^{III} or DMA^{V} for 24 hours in 2D culture. AS3MT was expressed in untreated control cells, and its expression tended to decrease in a concentration-dependent manner following both iAs^{III} and DMA^{V} treatment.
